# Genome-wide association study identified candidate genes for seed size and seed composition improvement in *M. truncatula*

**DOI:** 10.1038/s41598-021-83581-7

**Published:** 2021-02-19

**Authors:** Zhijuan Chen, Vanessa Lancon-Verdier, Christine Le Signor, Yi-Min She, Yun Kang, Jerome Verdier

**Affiliations:** 1 Univ Angers, Institut Agro, INRAE, IRHS, SFR QUASAV, 49000 Angers, France; 2 Shanghai Center for Plant Stress Biology, CAS Center for Excellence in Molecular Plant Sciences, Chinese Academy of Sciences, Shanghai, 200032 China; 3 Agroecologie, AgroSup Dijon, INRAE, Université Bourgogne Franche Comte, 21000 Dijon, France; 4 Noble Research Institute, LLC, Ardmore, OK 73401 USA; 5 Present Address: USC 1422 GRAPPE, INRAE, Ecole Supérieure d’Agricultures, SFR 4207 QUASAV, 55 rue Rabelais, 49100 Angers, France; 6 Present Address: Centre for Biologics Evaluation, Biologics and Radiopharmaceutical Drugs Directorate, Health Canada, Ottawa, ON K1A 0K9 Canada

**Keywords:** Agricultural genetics, Plant genetics

## Abstract

Grain legumes are highly valuable plant species, as they produce seeds with high protein content. Increasing seed protein production and improving seed nutritional quality represent an agronomical challenge in order to promote plant protein consumption of a growing population. In this study, we used the genetic diversity, naturally present in *Medicago truncatula*, a model plant for legumes, to identify genes/loci regulating seed traits. Indeed, using sequencing data of 162 accessions from the *Medicago* HAPMAP collection, we performed genome-wide association study for 32 seed traits related to seed size and seed composition such as seed protein content/concentration, sulfur content/concentration. Using different GWAS and postGWAS methods, we identified 79 quantitative trait nucleotides (QTNs) as regulating seed size, 41 QTNs for seed composition related to nitrogen (i.e. storage protein) and sulfur (i.e. sulfur-containing amino acid) concentrations/contents. Furthermore, a strong positive correlation between seed size and protein content was revealed within the selected *Medicago* HAPMAP collection. In addition, several QTNs showed highly significant associations in different seed phenotypes for further functional validation studies, including one near an RNA-Binding Domain protein, which represents a valuable candidate as central regulator determining both seed size and composition. Finally, our findings in *M. truncatula* represent valuable resources to be exploitable in many legume crop species such as pea, common bean, and soybean due to its high synteny, which enable rapid transfer of these results into breeding programs and eventually help the improvement of legume grain production.

## Introduction

Legume seeds are an important source to provide human food and animal feed. The high contents in proteins and carbohydrates, as well as fibers and minerals in legumes are an essential component of human diets ^1^. With the world population growing and the increasing need of plant proteins, producing highly nutritious seeds with high protein content, essential amino acids and minerals is in great demand.

Compared to grains, legume seeds have naturally high protein contents; however, they are deficient in sulfur-containing amino acids and have lower concentrations of certain dietary minerals such as Fe, Ca and Zn compared to animal proteins^2^. Increasing seed protein production and improving seed nutritional quality have been a challenge in the agronomic field.

The existing natural diversity of legume could help identify key molecular players in achieving these challenges by understanding its underlying molecular mechanisms and by identifying molecular markers. *Medicago truncatula* is a Mediterranean originated plant and has been a model plant of legumes from 1990^3,4^. Its genome was sequenced and has still been under development with a recent fifth release^5^.

Several quantitative trait loci (QTL) analyses have been performed in *M. truncatula* to identify loci affecting seed protein and mineral compositions^6,7^. Nevertheless, QTL identification depends on mapping population genetics of a few parents limited its use in exploratory genetic approach. Genome-wide association studies (GWAS) use a broad panel of natural accessions with high genetic diversity and could overcome QTL analysis limitations^8^. Nowadays, GWAS has become a useful approach to explore the genetics of natural accessions and agronomic traits. A *Medicago* HAPMAP collection of over 200 natural accessions has been developed, which contains several millions of single nucleotide polymorphisms (SNPs)^9^. This *Medicago* GWAS panel has been successfully employed to identify candidate loci/genes associated with various agronomic traits^4^ such as seed protein composition^7^.

In this study, we performed GWAS focusing on seed traits related to seed size and seed composition using 162 accessions from the *M. truncatula* HAPMAP collection. Moreover, we performed association studies using both single and multi-locus models as well as several postGWAS analyses in order to identify potential loci/genes that could be involved in seed nutritional qualities in *M. truncatula*.

## Results

### Phenotypic evaluation of seed traits among the HAPMAP seed collection

We evaluated the phenotypic variation of 162 *Medicago* accessions on 16 seed traits regarding seed size and composition, plus 16 additional traits related to seed mineral composition in a subset of 88 accessions. Seed size was determined by weight measurement, area, perimeter, length (called ‘majellipse’ for major axis of ellipse) and width (called ‘minellipse’ for minor axis of ellipse)^10^. Seed color variations (called CH1, CH2 and CH3) potentially reflected the secondary metabolite composition in the seed coat. Global seed composition was characterized including carbon, hydrogen, nitrogen and sulfur percentages (w/w) (called %C, %H, %N, %S). From these concentration values of nitrogen and sulfur, we estimated the nitrogen and sulfur contents per seed of each accession based on individual seed weights (traits called N Content and S Content and expressed in milligram per seed). Nitrogen concentration/content is a good indicator of the global protein content in seed and is commonly used for total protein determination in food products. Indeed, a predefined coefficient factor, Jones Factor^11^, is used to convert the nitrogen concentration into total protein content. This coefficient is 6.25, but might vary between species and plant tissues. We also calculated the ratio between carbon and nitrogen (C/N), which corresponds to a global seed composition estimation. Sulfur concentrations/contents were also characterized, which reflected high-quality storage proteins. Indeed, legume seeds generally have a low level of sulfur-containing amino acids, which were shown to be tightly regulated by plant sulfur status^12,13^. Finally, other minerals (i.e. macro- and micro-elements) were quantified in seeds from a subset of 88 accessions. Concentrations of macro- (P, K, Mg, Ca, Na) and micro- (Fe, Mn, Zn, Cu, Mo, Co, Ni, V) elements were determined in mature seeds. All phenotypic values for the analyzed accessions are provided in the Supplemental Table S1.

### Phenotypic diversity and correlation between seed traits and Impact of geographical location

A wide range of phenotypic variation was observed among the different accessions tested (Supplementary Figure S1 and Supplemental Table S1) with a coefficient of variation (CV) ranging from 1% for the most stable traits such as carbon and hydrogen concentrations, to 84% for Fe concentration. Other seed traits showed a high variability such as seed weight, N content and S content with CVs around 20%. In general, seed mineral concentrations showed the highest phenotypic diversity with Fe, Zn and Na displaying higher CV values. All the phenotypic values and CVs are provided in Supplementary Table S1.

**Figure 1:**
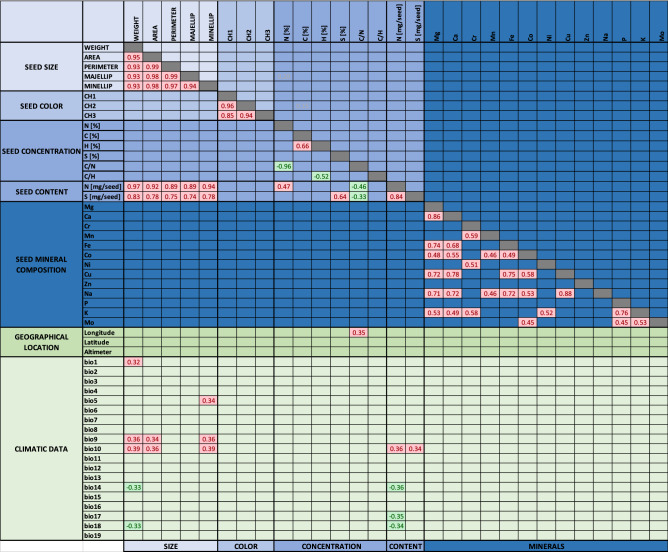
Correlation matrix between *Medicago* seed traits, and in relation to their geographical locations and climatic data. Only Pearson correlation coefficients (PCC) with adjusted p-values below 5% are indicated after BH procedure to control false discovery rate. Red color indicates PCC above 0.2 and green color indicates PCC below − 0.2. Longitude is expressed in degrees with negative degrees representing west and positive degrees representing east. Latitude is also expressed in degrees with negative degrees representing south and positive degrees representing north. Climatic data are from WorldClim. *BIO1* annual mean temperature, *BIO2* mean diurnal range, *BIO3* isothermality, *BIO4* temperature seasonality, *BIO5* max temperature of warmest month, *BIO6 *min temperature of coldest month, *BIO7* temperature annual range, *BIO8* mean temperature of wettest quarter, *BIO9* mean temperature of driest quarter, *BIO10* mean temperature of warmest quarter, *BIO11* mean temperature of coldest quarter, *BIO12* annual precipitation, *BIO13* precipitation of wettest month, *BIO14* precipitation of driest month, *BIO15* precipitation seasonality, *BIO16* precipitation of wettest quarter, *BIO17* precipitation of driest quarter, *BIO18* precipitation of warmest quarter, *BIO19 *precipitation of coldest quarter.

Due to the availability of geographical locations of each accession origin, we allocated different accessions to three geographical values (i.e. longitude, latitude, altimeter) and 19 bioclimatic values obtained from the WorldClim database (http://worldclim.org). These bioclimatic values (called BIO1 to BIO19) mainly represent temperature and rainfall values measured monthly, quarterly or annually (see details in Fig. 1 legend). A global correlation analysis was performed to identify correlations between seed phenotypic traits themselves and with their geographical and bioclimatic values (Fig. 1). Results showed that all seed traits related to seed size (i.e. weight, area, perimeter, minellipse and majellipse) were highly correlated (Pearson coefficient correlation, PCC > 0.9), which validated the accuracy of our measurements. Similar results were obtained for seed color values (i.e. PCC > 0.85 for CH1, CH2, CH3).

Regarding seed content, we observed that nitrogen and sulfur contents were also highly correlated with seed size traits (PCC > 0.89 for N content and 0.74 for S content), which suggested that variations in seed content were predominantly determined by seed size. Regarding mineral composition in seeds, we observed positive correlations between concentrations of some elements such as Ca, Mg, Fe, Cu and Na (PCC > 0.7) but also between the macro-elements P and K (PCC > 0.75, Fig. 1).

With the addition of the geographical values, we observed a moderate positive correlation between accession longitudes and seed C/N ratio (see the legend in Fig. 1), which indicated that accessions collected from the East tended to have higher C/N ratio (i.e. less nitrogen). To explain this difference, we also observed moderate positive correlations (PCC > 0.35) between seed size, seed contents (N and S) and temperature (i.e. BIO 9, 10), and at the opposite moderate negative correlations (PCC < -0.3) between seed weight, N content and precipitations (i.e. BIO 14, 17, 18). The integration of the bioclimatic data suggested that temperature and precipitation played an important role in accession adaptability to final seed size determination, with outcome in sulfur and nitrogen contents.

### Genome-wide association analysis of seed traits

**Figure 2:**
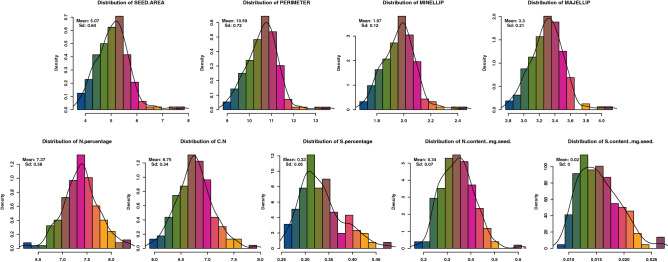
Distribution histograms of seed size and composition phenotypes in different Medicago accessions. Corresponding distribution curves are indicated on histograms. Different x-axes represent the corresponding values of the phenotypes.

In order to perform genome-wide association analysis, we first, used the Box-Cox procedure^14^ to estimate the appropriate lambda to transform our phenotypic data and, therefore, validate the assumption of normality required when performing GWAS prediction. Out of the 32 measured seed phenotypes, 26 traits were normalized using respective lambdas to finally display a normal distribution according to Shapiro–Wilk test (Fig. 2, Supplementary Table S1 and Supplementary Figure S1). However, six seed traits corresponding to the perimeter, CH1, %C, %H, C/H ratio and Arsenic (As) concentration were discarded from subsequent GWA analyses since, even after transformation, these traits did not reach normality.

In this study, two different models for genome-wide association predictions were applied to normalized phenotypes: a classical single-locus mixed linear model (EMMA^15^) with kinship and population structure as inputs, and a multi-locus model (FarmCPU^16^) with correction of population structure. When performing the multi-locus FarmCPU model, we observed QQ plots with a better fit between the expected and observed results following the expected null-hypothesis distribution of p-values (Supplementary Figure S2). These QQ plots reflected that most of the tested SNPs have no significant p-values, except for a few SNPs that have a strong and significant effect. Moreover, QQ plots obtained after performing the EMMA algorithm generally showed a curve corresponding to observed results below the theoretical curve (i.e. deflated curve), which suggested that this model was not appropriate for this association study. Regarding the Manhattan plots obtained from different models, we also observed differences between EMMA and FarmCPU (Supplementary Figure S2). In general, we obtained less background noise with FarmCPU, with more precise location and lower p-values of SNPs than the ones obtained from Mixed Linear Model (MLM), especially when statistical analysis showed highly significant SNPs. Manhattan plots obtained from MLM displayed broader “peaks” made of multiple significant SNPs (i.e. SNP clusters). Overall, we note that most of the highest significant SNPs were identified in both methods but FarmCPU provided more power detection and accuracy to identify quantitative trait nucleotides (QTNs) (Supplementary Figure S2). Therefore, we decided to focus on the multi-locus mixed model with FarmCPU in the subsequent analyses. All results (Manhattan and QQ plots) obtained from FarmCPU in this study are provided as Supplementary figures S3-S7. Moreover, gwas files directly readable on any genome browsers such as the web-accessible JBrowse^17^ or desktop genome viewer such Integrative Genome Viewer (IGV^18^) are also provided as Supplemental Tables S2-S5.

As previously described, we observed two contrasting situations regarding association studies and their resulting Manhattan plots: identification of highly significant QTNs with clear genomic location and identification of clusters of SNPs indicating associated loci. As preliminary results of these analyses, we clearly identified highly significant QTNs associated with seed size (Supplementary Figure S3) and seed composition (Supplementary Figure S4) present on several chromosomes. For instance, we observed five, six, four and six QTNs highly associated respectively with seed area, seed length, seed width and seed weight with a -log_10_(p-value) > 10 (i.e. p-value < 10^–10^). Regarding seed color (Supplementary Figure S5) and seed mineral concentrations (Supplementary Figure S6, S7), QTN p-values were significantly lower and nearer to background noise, which allowed only identification of specific genomic regions (i.e. SNP clusters), rather than highly significant individual QTNs.

**Figure 3:**
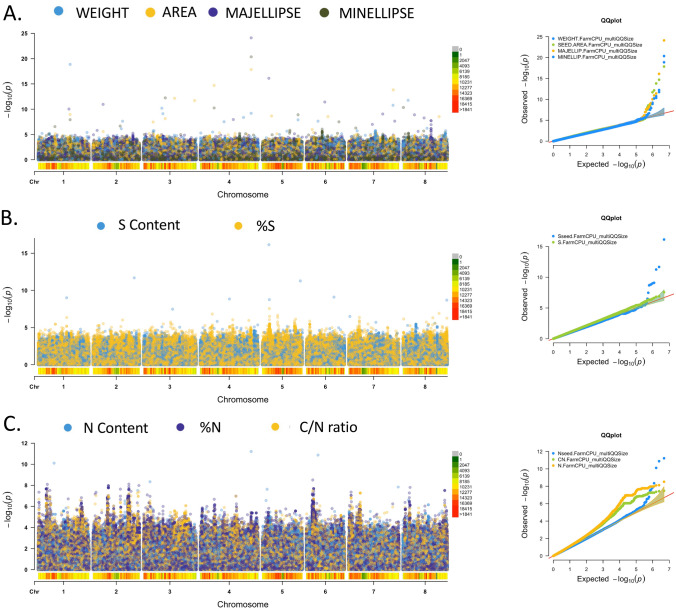
Genome-wide association studies of the *Medicago* seed traits with Manhattan plots and QQ plots obtained from FarmCPU. **(A)** Combination of association studies regarding seed size (weight, area, majellipse, minellipse). **(B)** Combination of association studies regarding seed sulfur content (mg/seed) and sulfur concentration (%, w/w). **(C)** Combination of association studies regarding seed protein content (nitrogen content (mg/seed); nitrogen concentration (%, w/w); carbon/nitrogen ratio).

To identify relevant QTNs, we combined association results from highly correlated seed traits. For instance, we combined FarmCPU results from weight, area, majellipse and minellipse (Fig. 3a) and identified common QTNs between seed size traits such as MtrunA17Chr4_56801315 on Chromosome 4. Interestingly, this QTN showed high p-values with all four seed size traits (10^–18^, 10^–25^, 10^–21^, 10^–10^ with respective area, majellipse, minellipse and weight), suggesting a reliable QTN regulating seed size. This QTN is located within the genomic sequence encoding for a protein containing an RNA binding motif (gene ID MtrunA17Chr4g0065741). Another potentially reliable QTN (MtrunA17Chr1_35506650) was identified from three different seed size phenotypes with highly significant p-values of 10^–9^, 10^–8^, 10^–19^ for area, minellipse and weight, respectively. This QTN located on chromosome 1, closely related to a genomic sequence encoding a WD40-LIKE transcription factor (gene ID MtrunA17Chr1g0185101).

Similarly, we compared association studies between sulfur content and sulfur concentration to identify four major QTNs shared between these two traits with low p-values (Fig. 3b). MtrunA17Chr1_31627600 on chromosome 1, located within the coding sequence of the EXPORTIN5 protein (MtrunA17Chr1g0180461) closely related to Arabidopsis HASTY1 protein, which was shown to act as a nucleocytoplasmic transporter involved in the nuclear export of small RNAs^19^. MtrunA17Chr4_32623172 in chromosome 4, located in a chromosomic region rich in transposable elements. MtrunA17Chr5_8051955 present in chromosome 5 and is close to a gene encoding a salicylate methyltransferase (SAMT, MtrunA17Chr5g0404631), which catalyzes the methylation of salicylic acid with S-adenosyl-L-methionine to form methyl salicylate (MeSA), mainly in response to stress^20^. MtrunA17Chr8_48959923 on chromosome 8, located in the promoter region of a gene encoding a histidine kinase (MtrunA17Chr8g0392301).

Regarding nitrogen composition, we compared association studies between nitrogen concentration, nitrogen content and CN ratio in seeds (Fig. 3c). Following this experiment, it was more difficult to identify clear QTNs such as the N concentration and the CN ratio result showed more genomic regions that individual and distinct QTNs associated with these phenotypes. However, it appeared that regions mainly located on chromosomes 1, 2, 6 and 8 showed strong associations between seed nitrogen composition and different accession polymorphisms, which suggested that these regions could play a role in seed nitrogen composition. Moreover, some particular QTNs were highly relevant for further analyses and indicated in Table 1. For instance, first, we identified a highly significant QTN (MtrunA17Chr6_7310002) associated with both protein concentration and C/N ratio, which is closely located to a genomic sequence encoding a putative amino acid transporter (MtrunA17Chr2g0333321). Second, we also identified a highly significant p-value for the QTN MtrunA17Chr4, which was already identified in the four seed size traits, in the N content association study. This result was predictable due to the high PCC between seed size and nitrogen content, which suggested that this QTN could be a regulator of both traits, making this QTN a potentially interesting candidate to improve concomitantly seed size and seed protein content.

**Table 1:** Top five QTNs significantly associated with different seed size traits (i.e. weight, area, majellipse, minellipse) and seed compositions (S content, N content, %S, %N and C/N ratio). SNP/QTN names, positions and p-values are indicated from FarmCPU. Numbers of potential associated SNP(s) and putative causal genes are indicated from PLINK analysis. Gene expression in major *Medicago* plant organs, as well as tentative gene annotations are indicated. A more exhaustive list of highly significant QTNs related to all seed traits is provided as Supplementary Table S6, and complete lists of SNPs and their associated p-values are provided as Supplementary Tables S2 to S5.

	GWAS (FarmCPU)				LD and putative causal gene(s) (PLINK)		Expression (RNA-seq) TPM							Annotations
	Traits	Chromosome	SNP ID (Chr_position)	P-Value	Number of potential SNP in LD according to PLINK (including QTN)	Associated gene(s)	Pod	Blade	Flower	Nodule	Root	Root_all	Shoot_all	Description
Seed size	Area	4	MtrunA17Chr4_56799264	1.40E−18	1	MtrunA17Chr4g0065741	1.12	1.42	1.22	0.60	1.62	3.89	11.25	RNA-binding (RRM RBD RNP motif) family
	Area	4	MtrunA17Chr4_15564603	2.00E−15	1									
	Area	7	MtrunA17Chr7_49921035	1.53E−14	1	MtrunA17Chr7g0267081	5.19	1.67	6.29	4.57	4.19	8.56	7.49	Probable CCR4-associated factor 1 homolog 11
	Area	3	MtrunA17Chr3_34669807	6.93E−13	1	MtrunA17Chr3g0112751	2.92	0.77	7.64	8.62	11.33	36.22	12.29	Hypothetical protein MTR_3g069670
	Area	3	MtrunA17Chr3_57001194	1.96E−12	1	MtrunA17Chr3g0143421								
	Area					MtrunA17Chr3g0143431	0.00	0.00	0.05	1.85	4.26	52.45	2.45	Syringolide-induced 14-1-1
	Area					MtrunA17Chr3g0143441	11.60	2.33	11.12	25.66	16.91	76.05	25.55	24-methylenesterol C-methyltransferase 2
	Majellip	4	MtrunA17Chr4_56801315	7.74E−25	1	MtrunA17Chr4g0065741	1.12	1.42	1.22	0.60	1.62	3.89	11.25	RNA-binding (RRM RBD RNP motif) family
	Majellip					MtrunA17Chr4g0065751	3.05	6.48	10.23	5.50	11.43	31.59	16.96	DUF21 domain-containing At4g14240-like
	Majellip	5	MtrunA17Chr5_7453281	7.83E−17	1									
	Majellip	6	MtrunA17Chr6_20728994	3.78E−12	1									
	Majellip	2	MtrunA17Chr2_11271424	1.10E−11	1	MtrunA17Chr2g0292281	0.00	0.00	0.00	0.00	0.00	0.45	0.11	Peroxidase family
	Majellip					MtrunA17Chr2g0292291	0.00	0.03	0.00	1.23	76.96	602.07	0.00	Peroxidase family
	Majellip	1	MtrunA17Chr1_34226006	9.20E−11	1	MtrunA17Chr1g0183471	2.04	0.56	8.05	5.46	4.05	3.49	2.62	Hypothetical protein MTR_1g069640
	Majellip					MtrunA17Chr1g0183481	2.44	1.39	4.30	6.04	3.33	0.00	0.00	unknown
	Minellip	4	MtrunA17Chr4_56799264	4.31E−21	1	MtrunA17Chr4g0065741	1.12	1.42	1.22	0.60	1.62	3.89	11.25	RNA-binding (RRM RBD RNP motif) family
	Minellip	3	MtrunA17Chr3_24325917	5.75E−13	1	MtrunA17Chr3g0099571	0.00	0.00	0.04	0.78	4.94	8.94	0.06	Disease resistance (CC-NBS-LRR class) family
	Minellip					MtrunA17Chr3g0099581	0.06	0.00	0.00	0.75	2.95	7.38	0.27	Probable disease resistance At4g27220
	Minellip					MtrunA17Chr3g0099591	0.86	3.27	1.95	0.08	0.33	1.03	14.96	Disease resistance (CC-NBS-LRR class) family
	Minellip	8	MtrunA17Chr8_628023	4.46E−11	1	MtrunA17Chr8g0334971	0.00	0.00	0.00	0.00	0.00	0.00	0.00	DUF247 domain
	Minellip	4	MtrunA17Chr4_15564603	5.65E−11	1									
	Minellip	5	MtrunA17Chr5_39987332	1.23E−09	1	MtrunA17Chr5g0441651	4.40	2.36	22.68	16.31	11.53	33.46	14.26	Dihydropyrimidinase
	Minellip					MtrunA17Chr5g0441661	0.58	0.00	39.34	0.00	0.00	0.00	0.00	RPM1-interacting 4 (RIN4) family
	Minellip					MtrunA17Chr5g0441671	11.02	4.34	26.25	30.95	42.28	163.92	77.69	Splicing factor 3B subunit 6
	Minellip					MtrunA17Chr5g0441681								
	Minellip					MtrunA17Chr5g0441691	0.58	0.66	7.45	0.18	0.51	0.57	2.74	Calcium-dependent kinase 17
	Minellip					MtrunA17Chr5g1024447								
	Weight	1	MtrunA17Chr1_35506650	1.33E−19	1	MtrunA17Chr1g0185101	2.79	2.49	4.33	12.71	12.22	22.03	4.59	BEACH domain-containing lvsA
	Weight	8	MtrunA17Chr8_5914802	1.74E−12	1									
	Weight	7	MtrunA17Chr7_49559389	1.66E−11	1	MtrunA17Chr7g0266521	202.23	7.53	100.55	140.68	41.92	39.45	24.23	Transmembrane protein, putative
	Weight					MtrunA17Chr7g0266531	70.18	1.94	27.86	52.11	14.60	17.34	10.28	Transmembrane protein, putative
	Weight					MtrunA17Chr7g0266541	43.90	4.20	9.31	30.74	0.00	0.35	0.00	Hypothetical protein MtrDRAFT_AC150442g27v2
	Weight					MtrunA17Chr7g0266551	0.00	0.00	0.00	0.85	0.00	0.41	0.00	Hypothetical protein MTR_7g104915
	Weight					MtrunA17Chr7g0266561	9.53	3.25	13.63	10.84	14.31	30.07	19.35	Probable small nuclear ribonucleo G
	Weight	3	MtrunA17Chr3_20500592	6.27E−11	1									
	Weight	4	MtrunA17Chr4_56799264	6.39E−10	1	MtrunA17Chr4g0065741	1.12	1.42	1.22	0.60	1.62	3.89	11.25	RNA-binding (RRM RBD RNP motif) family
Seed composition	S content	5	MtrunA17Chr5_7518926	7.21E−17	1	MtrunA17Chr5g0403771	0.02	1.48	0.59	0.16	0.18	2.86	1.68	Vicilin-like antimicrobial peptides 2-2
	S content					MtrunA17Chr5g0403781	5.98	11.36	8.86	2.58	2.27	8.34	94.20	Probable phosphatase 2C 80
	S content	2	MtrunA17Chr2_45988522	2.09E−12	1	MtrunA17Chr2g0326151	0.21	0.38	1.99	0.54	0.13	0.50	0.70	Pre-mRNA-processing-splicing factor 8
	S content					MtrunA17Chr2g0326161	0.00	0.00	0.00	0.00	0.00	0.42	0.00	Allergen gly M Bd 28 kDa
	S content	5	MtrunA17Chr5_42555471	5.47E−12	1	MtrunA17Chr5g0445531	0.00	0.00	52.16	0.00	0.00	0.00	0.08	Cytochrome P450 family 71
	S content					MtrunA17Chr5g0445541	0.00	0.00	0.00	0.00	0.00	0.00	0.00	Cytochrome P450 family 71
	S content					MtrunA17Chr5g0445551	0.00	5.07	0.00	4.75	0.00	0.00	0.00	Cytochrome P450 family 71
	S content					MtrunA17Chr5g0445561	0.00	0.00	0.00	0.00	0.00	0.17	0.00	Zinc C3HC4 type (RING finger)
	S content	6	MtrunA17Chr6_30934715	8.09E−10	1									
	S content	1	MtrunA17Chr1_31627600	9.84E−10	1	MtrunA17Chr1g0180461	0.00	0.00	0.00	0.00	0.00	0.00	0.00	HASTY 1
	S content					MtrunA17Chr1g0180471	0.00	0.20	0.00	0.00	0.00	0.00	0.64	HASTY 1
	S content					MtrunA17Chr1g0180481	0.07	0.12	0.00	0.00	0.00	0.00	1.09	HASTY 1
	%S	5	MtrunA17Chr5_8051955	2.76E−08	3	MtrunA17Chr5g0404631	0.00	0.04	57.28	0.24	0.26	5.92	0.23	Salicylate O-methyltransferase
	%S					MtrunA17Chr5g0404641	0.00	0.00	0.11	0.83	0.00	0.83	0.33	Heavy-metal-associated domain
	N content	4	MtrunA17Chr4_56799264	6.14E−12	1	MtrunA17Chr4g0065741	1.12	1.42	1.22	0.60	1.62	3.89	11.25	RNA-binding (RRM RBD RNP motif) family
	N content	6	MtrunA17Chr6_13176638	1.33E−11	1									
	N content	1	MtrunA17Chr1_17611945	7.77E−11	1	MtrunA17Chr1g0168711	34.54	36.56	59.95	28.95	53.04	113.94	152.11	3-isopropylmalate dehydratase large subunit-like
	N content	3	MtrunA17Chr3_7317464	4.52E−09	1	MtrunA17Chr3g0085931	0.03	0.00	0.00	2.64	3.37	8.18	0.06	NBS-LRR type disease resistance
	N content					MtrunA17Chr3g0085941	0.00	0.00	0.00	0.00	0.09	0.00	0.00	Cytochrome C biogenesis ccsA
	N content	7	MtrunA17Chr7_55339972	1.45E−08	1	MtrunA17Chr7g0275391	0.97	4.94	2.71	0.45	1.11	3.07	12.83	Copper-transporting ATPase chloroplastic-like isoform X1
	%N	6	MtrunA17Chr6_7310002	3.07E−09	1	MtrunA17Chr6g0457641	0.00	0.00	0.00	0.00	0.00	0.00	0.00	Cytochrome C biogenesis ccsA
	%N					MtrunA17Chr6g0457651	0.00	0.00	0.00	0.00	0.00	0.00	0.00	Transmembrane protein, putative
	%N					MtrunA17Chr6g0457661	2.52	1.51	5.22	5.25	3.84	21.16	13.28	Zinc transporter 5-like isoform X2
	%N					MtrunA17Chr6g0457671	4.90	2.09	9.93	6.56	8.10	15.08	9.78	Zinc transporter 5
	%N	2	MtrunA17Chr2_39997412	7.52E−09	27	MtrunA17Chr2g0318301	4.50	1.66	5.76	11.51	8.44	25.57	15.99	Receptor kinase THESEUS 1
	%N					MtrunA17Chr2g0318311	0.00	0.00	0.07	0.00	1.92	4.68	0.05	Root cap late embryogenesis
	%N					MtrunA17Chr2g0318321	0.00	0.80	1.10	0.00	0.00	0.00	0.00	RNA polymerase beta partial (chloroplast)
	%N					MtrunA17Chr2g0318331	0.00	0.00	0.00	0.00	0.00	0.00	0.00	Receptor kinase THESEUS 1
	%N					MtrunA17Chr2g0318341	0.00	0.06	0.00	0.15	67.70	88.60	0.00	Root cap late embryogenesis
	%N					MtrunA17Chr2g0318351	28.50	12.81	54.94	39.90	53.20	110.12	69.26	Hypothetical protein MTR_2g080270
	%N					MtrunA17Chr2g0318361	0.00	0.00	0.00	0.00	0.00	0.83	0.00	Hypothetical protein MTR_2g080280
	%N					MtrunA17Chr2g0318371	4.61	28.32	12.88	1.56	6.66	7.97	66.65	Transmembrane protein, putative
	%N					MtrunA17Chr2g0318381	0.00	0.00	0.00	0.00	0.00	0.00	0.00	Pentatricopeptide repeat-containing At1g20230-like
	%N	1	MtrunA17Chr1_10142836	8.00E−09	4	MtrunA17Chr1g0159441	7.70	8.39	21.83	25.52	29.94	65.42	22.63	Transcriptional corepressor SEUSS
	%N					MtrunA17Chr1g0159451	2.40	3.50	7.74	6.58	10.84	19.75	9.76	Small RNA degrading nuclease 5
	%N	2	MtrunA17Chr2_51147031	1.03E−08	1	MtrunA17Chr2g0333321	1.01	0.71	3.17	3.07	2.20	3.40	2.02	Probable sodium-coupled neutral amino acid transporter 6
	%N	2	MtrunA17Chr2_17222898	1.07E−08	4	MtrunA17Chr2g0299211								
	%N					MtrunA17Chr2g0299221	0.00	0.00	0.00	1.87	0.00	0.00	0.00	Little zipper
	%N					MtrunA17Chr2g0299231	0.00	0.00	0.00	0.20	0.00	0.00	0.00	Hypothetical protein MTR_2g039220
	%N					MtrunA17Chr2g0299241	0.00	0.00	0.31	9.33	0.63	0.00	0.00	Nodule-specific Glycine Rich Peptide
	%N					MtrunA17Chr2g0299251	0.00	0.00	0.00	0.30	0.00	0.00	0.00	NA
	%N					MtrunA17Chr2g0299261	0.08	0.00	0.34	274.55	0.23	0.05	0.00	Nodule-specific Glycine Rich Peptide
	%N					MtrunA17Chr2g0299271	41.21	10.74	67.19	90.53	54.34	111.62	71.08	WD-40 repeat-containing MSI4
	CN ratio	6	MtrunA17Chr6_7310002	3.36E−08	1	MtrunA17Chr6g0457641	0.00	0.00	0.00	0.00	0.00	0.00	0.00	Cytochrome C biogenesis ccsA
	CN ratio					MtrunA17Chr6g0457651	0.00	0.00	0.00	0.00	0.00	0.00	0.00	Transmembrane protein, putative
	CN ratio					MtrunA17Chr6g0457661	2.52	1.51	5.22	5.25	3.84	21.16	13.28	Zinc transporter 5-like isoform X2
	CN ratio					MtrunA17Chr6g0457671	4.90	2.09	9.93	6.56	8.10	15.08	9.78	Zinc transporter 5
	CN ratio	1	MtrunA17Chr1_10142836	3.75E−08	4	MtrunA17Chr1g0159441	7.70	8.39	21.83	25.52	29.94	65.42	22.63	Transcriptional corepressor SEUSS
	CN ratio					MtrunA17Chr1g0159451	2.40	3.50	7.74	6.58	10.84	19.75	9.76	Small RNA degrading nuclease 5
	CN ratio	2	MtrunA17Chr2_51147031	4.35E−08	1	MtrunA17Chr2g0333321	1.01	0.71	3.17	3.07	2.20	3.40	2.02	Probable sodium-coupled neutral amino acid transporter 6
	CN ratio	7	MtrunA17Chr7_13129833	5.48E−08	30	MtrunA17Chr7g0227971	0.00	0.18	0.00	0.28	0.00	0.40	0.92	Nucleoporin GLE1
	CN ratio					MtrunA17Chr7g0227981	4.76	3.46	18.54	13.53	13.39	18.60	19.11	N-terminal glutamine amidohydrolase
	CN ratio					MtrunA17Chr7g0227991	0.00	0.00	0.18	0.00	0.00	0.00	0.00	Subtilisin-like serine endopeptidase family
	CN ratio	2	MtrunA17Chr2_49391628	5.58E−08	1	MtrunA17Chr2g0330811	0.55	1.00	2.15	1.25	0.77	3.27	8.91	Chloroplastic group IIA intron splicing facilitator chloroplastic isoform X1
	CN ratio					MtrunA17Chr2g0330821	0.45	0.72	1.86	1.74	3.10	6.44	2.60	Heat shock transcription factor A8
	CN ratio					MtrunA17Chr2g0330831	0.00	0.00	8.21	6.70	3.44	2.69	1.71	NA
	CN ratio					MtrunA17Chr2g0330841	0.33	0.21	0.83	1.34	0.74	5.49	2.23	Heat stress transcription factor A-5-like

Regarding seed color and seed mineral concentrations, several loci were identified by combining results from CH2 and CH3 and from all macro- and micro-element concentrations. However, no major QTNs (i.e. p-values > 10^–10^) and precise location of SNP clusters were identified. This absence of highly significant QTNs regarding seed mineral concentrations could be explained by the small population size used in this specific analysis (i.e. subset of 88 accessions).

### PostGWAS analyses to identify putative causal genes

To shorten the list of candidate QTNs, we used p-value threshold of 10^–7^ when association studies displayed high SNP power detection such as seed size and seed composition phenotypes, and a p-value threshold of 10^–5^ when association analyses displayed low SNP power detection such as seed color and seed mineral concentrations. Then, the linkage disequilibrium (LD) was considered to identify putative causal genes associated with selected QTNs. Considering that in the *Medicago* HAPMAP collection, the average LD decay was determined around 15kb^21^, we performed genome-wide correlations between selected SNPs present within this genomic range (i.e. ± 15 kb from QTNs) using PLINK^22^. A threshold correlation of 0.7 was used to identify SNPs potentially in LD within these genomic regions. From this analysis, we established a list of SNPs correlated to the selected QTNs due to LD and therefore potential causal genes. From this list, we revealed 56 putative causal genes related to the 34 QTNs with highly significant p-values that are potentially involved in seed size determination, 123 putative causal genes related to the 56 QTNs potentially involved in seed composition, 90 putative causal genes related to the 45 QTNs potentially involved in seed color and 906 putative causal genes related to the 597 QTNs potentially involved in seed mineral composition (Table 1 and Supplementary Table S1). Due to the relatively low number of ecotypes used for the QTN identification related to seed nutritional composition, which might affect the statistical accuracy of the study, we decided to provide these results as supplementary data but we will not analyze them further.

In order to identify functional classes that could be involved in regulating these different seed phenotypes, we performed over-representation gene ontology (GO) analyses with corresponding lists of putative causal genes for each phenotype (Table 2). Interestingly, we observed that list of putative causal genes regulating seed size were enriched in GO terms related to the U12-type spliceosomal complex (GO:0005689). Similarly, using list of putative causal genes regulating seed protein content/concentration, we observed enrichment of genes with GO terms referring to nutrient reservoir activity (GO:0045735), amino acid transport (GO:0015171, GO:0003333) and oxalate metabolic pathway (GO:0033609, GO:0046564), which are all functional classes closely related to biosynthesis or transport of amino acids^23^. From putative genes regulating the seed color, we revealed that the GO terms referring to flavonoid biosynthesis were enriched (i.e. GO:0080043, GO:0080044, GO:0052696), and it has been shown that, indeed, flavonoid composition/concentration is closely related to seed coat color^24^. Finally, we observed enrichment of the GO term related to the protein amino acid autophosphorylation (GO:0046777) concerning genes potentially regulating mineral concentrations, which was less intuitive and presumably has indirect relations.

**Table 2:** Enrichment analysis of Gene Ontology (GO) terms on putative causal genes regulating different seed traits (i.e. size, composition and color).

ID	Description	p value	q value	Count
**Size**				
GO:0005689	U12-type spliceosomal complex	0.0004	0.0267	2
**Composition**				
GO:0045735	Nutrient reservoir activity	0.0000	0.0004	5
GO:0033609	Oxalate metabolic process	0.0000	0.0004	4
GO:0046564	Oxalate decarboxylase activity	0.0000	0.0004	4
GO:0030145	Manganese ion binding	0.0001	0.0011	4
GO:0015171	Amino acid transmembrane transporter activity	0.0002	0.0019	4
GO:0003333	Amino acid transmembrane transport	0.0002	0.0023	4
**Color**				
GO:0080043	Quercetin 3-*O*-glucosyltransferase activity	0.0003	0.0054	4
GO:0080044	Quercetin 7-*O*-glucosyltransferase activity	0.0003	0.0054	4
GO:0052696	Flavonoid glucuronidation	0.0004	0.0054	4

Enrichment p-values from hypergeometrical tests and q-values from Bonferroni corrections are indicated, as well as the number of genes annotated (count). Results were generated with R package “ClusterProfiler”.

In order to identify potential specific regulator of seed traits, we also focused on seed expression specificity and compared list of genes specifically expressed in seeds and pods with our list of candidate causal genes related to seed traits. Expression analysis in different *Medicago* plant organs was performed using publicly available information. To compare with our data, we mapped these reads to the *Medicago* genome version 5^5^ and quantified transcript expression using the Salmon pipeline^25^. Out of 44,473 transcripts in the *Medicago* genome (v5). 375 were identified as specifically or preferentially expressed in pods/seeds (Supplementary Table S7). After combining a list of seed-specific genes and our list of putative causal genes from GWA studies, we revealed two seed-specific genes potentially regulating seed nitrogen concentration: a zinc-finger transcription factor (MtrunA17Chr7g0217321) and a CAAT-Binding Transcription factor (CBF, MtrunA17Chr2g0318461), and eight seed-specific genes potentially regulating various mineral concentrations in seeds (Supplementary Table S6).

## Discussion

### Improving seed protein content in *M. truncatula* seeds by increasing seed size

Grain legumes play a key role in providing plant proteins for food and feed. Therefore, understanding how to increase seed protein content and to produce storage proteins with high nutritional values (i.e. containing essential amino acid and sulfur-containing amino acids) represents a technological breakthrough that has to be yet overcome. In this study, we observed significant genetic variabilities regarding seed traits such as size, nitrogen content (i.e. storage protein content) and sulfur content (i.e. sulfur-containing amino acid content), which makes the *Medicago* HAPMAP collection a great tool to improve these agronomical traits. Interestingly, our correlation matrix between these different seed traits within the Hapmap population revealed a strong correlation (PCC > 0.9) between seed size and protein content (Fig. 1), which suggested that increasing seed protein content could be directly achieved by increasing seed size. This hypothesis could, first, be confirmed by identification of colocalized QTLs of seed size and seed protein content in garden pea^26^, soybean^27^, Common Bean^28^ and cowpea^29^. In parallel, even if several genetic studies already highlighted genes controlling seed size, which generally act via regulation of mitotic activity in embryo and endosperm, such as *SBT1.1*^30^ and *DASH*^31^in *M. truncatula,* but also via regulation of cell elongation in endosperm and seed coat such as *ZHOUPI*^32^ and *TTG2*^33^ in *A. thaliana *(for review^34^). The hypothesis that increasing seed size would increase protein content is difficult to validate from literature because mutant lines displaying larger seeds were not tested for their protein contents and inversely, mutant lines affected in protein content were not tested for seed size. One exception is the gene *AP2* in Arabidopsis, which produced larger seeds in mutant plants combined with an increase in protein and fatty acid content^35^, which validate our hypothesis. Finally, numerous correlation analyses between seed size and protein content have been conducted on cereals and legumes but no general trend was observed. Indeed, even if several studies concluded about clear positive correlations between seed size and seed protein content in pigeon pea^36^, soybean^37^ and this study in *Medicago*, many others did not, suggesting genotype-environment effects. As mentioned earlier, these results are undoubtedly dependent on plant genetic background, favorable growth conditions and optimal agricultural practices. Indeed, in our study, we revealed that the geographical and bioclimatic origins of *Medicago* accessions played an important role in plant adaptation with correlations between seed size, seed content, temperature and precipitation during the reproductive phase (Table 1). These accessions showed a phenotypic adaptability to produce larger and higher seed protein content. Moreover, the variations of these traits within the same genetic backgrounds are also to consider as abiotic stress is known to affect proper seed development in *Medicago*^38^. Finally, one essential aspect to validate this positive correlation between seed size and protein content is the non-limiting nitrogen supply, which could be achieved via intensive nitrogen fertilization or via nitrogen fixation in legumes, which is still active during seed filling. In this study, we highlighted genes/loci potentially involved in seed size, but also in both seed size and seed protein content, which could potentially improve simultaneously seed nutritional values and agronomical performances, as it is already well documented that larger seeds tend to improve germination vigor and plantlet establishment (for review^39^).

### Efficiency of GWAS and post-GWAS algorithms

In the past 10 years due to the rapid development of genome sequencing technologies and phenotypic capacities, numerous genome-wide association studies (GWAS) have been performed in many species. This powerful tool is becoming a standard in forward genetic study to identify genes/loci controlling various traits. Its rapid development has been accompanied by the development of mainly two association study methodologies: classical single-locus GWAS methods based on General Linear Model (GLM) and Mixed Linear Model (MLM) (e.g. EMMA^15^; SUPER^40^), and recently developed multi-locus GWAS methods such as MLMM^41^, FASTmrEMMA^42^ and FarmCPU^43^. In the single-locus method, statistical tests are performed one locus at each time, whereas multi-locus methods consider the information of all loci simultaneously and consequently do not require false discovery rate correction, leading to higher QTN detection power^44^. In our study, we compared a single-locus method, EMMA, and a multi-locus method, FarmCPU, and we had two observations. (i) When association studies revealed highly significant candidate QTNs, FarmCPU (i.e. multi-locus method) resulted in more significant QTNs with lower p-values and more precise chromosome positions. Indeed, EMMA (i.e. the single-locus method) showed higher QTN p-values, closer to the background noise, which led to the identification of loci represented by broader “peaks” containing multiple significant SNPs (i.e. SNP clusters) in Manhattan plots, therefore more difficult to precisely locate on chromosomes (Figure S2). However, even if FarmCPU identified more significant QTNs with more precise locations, most of the highly significant QTNs were observed using both methods. (Figure S2A-B). (ii) When association studies did not reveal significant QTNs, single and multi-locus methods performed similarly (Figure S2C). In conclusion, from our study, it appeared that FarmCPU, the multi-locus method, globally performed better than the single-locus method, which explains why we focused on this method to identify candidate QTNs. Better performances of GWAS multi-locus models have also been observed in several other studies such as in Xu et al*.*^45^ related to starch properties in maize, Jaiswal et al*.*^46^ related to agronomic traits in wheat, and Li et al*.*^47^ related to fiber quality in Cotton, rendering these methods attractive for association studies.

### Potential regulation of seed size and protein content via RNA regulation

In order to determine reliable QTNs and mine for causal candidate genes controlling seed size and composition, we performed postGWAS analyses. First, we considered a 15 kb LD decay (r2 > 0.7), as determined in *Medicago* hapmap collection^21^, to identify associated SNPs due to LD. Then, depending on the association results, we used different approaches to refine candidate gene selection: combination of association results from correlated phenotypes to identify putative causal genes, use of over-representation analysis to identify key functional classes regulating phenotypes, and integration of transcriptomics.

Regarding seed size, we mined two highly significant QTNs associated with multiple seed size phenotypes by combining GWAS results of weight, area, majellipse and minellipse. First, MtrunA17Chr1_35506650, a QTN detected in three association studies (i.e. weight, minellipse and area), is near a gene encoding a WD40/BEACH domain protein (MtrunA17Chr1g0185101) (Table 1 and Supplemental Table S6). A potential ortholog of this gene in Arabidopsis, called SPIRRIG (SPI, AT1G03060), has been shown to be involved in cell morphogenesis via interaction with processing bodies (i.e. p-bodies)^48^, which is known to regulate mRNA processing during development or stress (for review^49^). In Arabidopsis, *spi* mutant lines displayed many developmental defects^50^ including reduced seed coat mucilage and plant growth impairment under salt stress^51^. Interestingly, the second QTN (MtrunA17Chr4_56801315) detected in all four association studies related to seed size was closely related with a gene encoding an RNA-binding domain (RBD, MtrunA17Chr4g0065741), which is also a gene involved in the regulation of RNA. RDB proteins belong to a large protein family, which are known to determine RNA fate from synthesis to degradation. Few of them have been functionally characterized and depending on their RNA targets, they could play tissue- and developmental stage-specific roles^52^. For instance, one of RDB protein family functionally characterized in Arabidopsis seed development is *SUPPRESSOR OF ABI3* (*SUA*, AT3G54230), which controls alternative splicing of the ABI3, a master regulator of seed development and maturation^53^. This QTN identified from several seed size association studies was also detected in association with the seed nitrogen content (Table 2), which indicated the important role of this gene in regulating both seed size and protein content.

This role of RNA processing/regulation to regulate seed size was further highlighted by the over-representation analysis of all highly significant QTNs associated with seed size, which revealed that the “U12-type spliceosomal complex” class was over-represented. This complex is part of the minor spliceosome, which plays a crucial role in splicing regulation of the rare U12 introns. It has been shown in Arabidopsis that homozygote mutant lines impaired in the U12 spliceosome complex displayed premature embryo abortion, whereas heterozygote mutants were defective for seed maturation, indicating an essential role of this complex during embryonic development^54^. Moreover, proper splicing and alternative splicing have been shown to be crucial in normal embryo formation (for review^55^) and embryo development, which is a key stage in controlling the final seed size.

## Methods

### *Medicago* plant accession and growth

Accessions from the HapMap germplasm collection were requested from the dedicated website (http://www.Medicagohapmap.org/hapmap/germplasm). Around 200 accessions were grown in growth chambers (20 °C/18 °C, 16 h photoperiod at 200 mmol m^−2^ s^−1^) until maturity. Mature seeds of 162 accessions were collected in sufficient quantity to perform different phenotyping experiments.

### Seed size and color determination

Individual seed weights of 162 accessions were estimated by weighting 50 seeds in triplicate using a precision balance at an accuracy ± 0.1 mg and displayed as mg per seed. To complete seed size phenotyping, image analyses were performed on 150 seeds of each accession using GrainScan software^10^ to automatically measure individual seed areas (i.e. pixel number, called “area”), seed perimeters (“perimeter”), seed lengths (“majellip”) and seed widths (“minellip”). These seed size parameters were averaged for each of the 162 accessions and used for the subsequent analyses. Image analysis also allowed us to determine seed color values using GrainScan, which measured three color channels (i.e. CH1, CH2, CH3) from raw RGB values, reflecting seed coat pigmentation.

### Seed composition analysis with elemental CHNS analyzer (162 accessions)

Seed composition was characterized using a CHNS elemental analyser, which measured the percentage (w/w) of carbon (C), hydrogen (H), nitrogen (N) and sulfur (S). Mature seeds were ground in liquid nitrogen and dried in an oven at 90 °C for 48 h. Then, triplicates of approximately 5 mg of powder were analyzed using an Elementar Vario Micro cube analyzer (Germany) using flash combustion of the sample based on the “Dumas” method. Concentrations of C, H, N, S were determined by the Elementar Vario software based on exact seed weights. From which, carbon–nitrogen ratios (C/N ratio) were calculated to provide an accurate overview of the global seed composition. Nitrogen and sulfur contents per seed for each accession (i.e. N content, S content) were calculated using average seed weights of each lot.

### Macro- and micro-element concentrations

A subset of 88 accessions was analyzed to determine elemental concentrations for P, K, Mg, Ca, Na, Fe, Mn, Zn, Cu, Mo, V, Co, Ni, Ti, As, Cr using Induced Coupled Plasma-Mass Spectrometry (ICP-MS, Perkin Elmer model NexION 300D). Seed powders were dried in a heating oven at 75 °C for overnight. Approximately 5 mg of seed powder were accurately weighed and transferred to a glass container with 3 ml of concentrated nitric acid (HNO_3_). After digestion for 15 min at 200 °C, deionized water was added to adjust the final volume to 10.0 ml and samples were injected into the ICP-MS for measurement. A blank sample containing 5% HNO_3_ was used for background subtraction. Concentrations (i.e. ppb or mg/L) of each element were calculated based on an internal standard mix (Perkin Elmer, ref. 9301721) and normalized according to a weight normalization procedure using the NexION software (Perkin Elmer).

### Correlation analysis

Correlation matrix was performed on averages of phenotype values. Each pairwise comparison was performed using Pearson correlation calculated using the complete pairwise correlation of the ‘corr.test’ function from the R package ‘psych’. P-values were adjusted using Benjamini-Hochberg (BH) to control false discovery rate and statistical significance threshold was set below 5% of adjusted p-values.

### Phenotype normality distributions

All traits were checked and transformed to reach normality as it is required to perform genome wide association studies. Box Cox algorithm^14^ was used to determine the appropriate transformation for each trait, and each trait was transformed separately according to the most suitable lambda values given by the Box Cox function implemented in the R package MASS^56^. After transformation, Shapiro–Wilk tests^57^ were performed to validate the normality and traits that did not reach normality were discarded of following GWAS analyses. Supplementary Table S1 provides seed trait values before and after Box Cox transformation, respective lambda values for each trait and corresponding p-values of the Shapiro–Wilk test after transformation.

### Genome-wide association studies and post-GWAS analyses

Single nucleotide polymorphisms (SNP) data were obtained by whole genome sequencing of the 262 *Medicago* accessions from the *M. truncatula* Hapmap project^9^. From the 6 million SNPs originally identified in *Medicago* genome version 4, 4,852,061 SNPs were successfully mapped to the fifth version of the *Medicago* genome (Mtv5^5^) and were used for subsequent analyses. The population structure and the kinship matrix used in this study were the same as previously described in Bonhomme et al*.*^58^ and le Signor et al*.*^7^, respectively. Two models were used to perform GWAS: (1) a classical single locus method using a mixed linear model called EMMA (Efficient Mixed-Model Association^15^ with the kindship matrix and the population structure as inputs; (2) a multi-locus model called FarmCPU (Fixed and random model Circulating Probability Unification^16^) with correction of the population structure, both with a statistical test p-value threshold of 1%. The Manhattan and quantile–quantile (QQ) plots were plotted using the R package rMVP (https://github.com/xiaolei-lab/rMVP). PostGWAS analysis was performed to correct for the linkage disequilibrium (LD) using PLINK algorithm^22^ with the “clump” function and the following options: clumb-kb-radius of 15, which represents the genomic range (in kilobases) to identify SNP in LD and clump-r2 of 0.7, which represents the r-squared threshold to identify correlation between SNPs. All GWAS result files were transformed into gwas files (Supplementary Tables S2 to S5) readable in web-application JBrowse^17^ containing the *M. truncatula* genome version 5 such as https://Medicago.toulouse.inra.fr/MtrunA17r5.0-ANR/ or in personal desktop genome viewer such as the freely available Integrative Genome Viewer (IGV^18^, http://software.broadinstitute.org/software/igv/). Over-representation analyses (ORA) of candidate genes were performed using ClusterProfiler package available in R using hypergeometrical test (p-values) with a Bonferroni correction (q-values)^59^.

### RNA-seq analysis in major plant organs

Expression of *Medicago* transcripts in major plant organs was determined from existing experiments. Sequenced short reads (i.e. raw fastq files) were downloaded from the Sequencing Read Archive (SRA, https://www.ncbi.nlm.nih.gov/sra) from different experiments and different *Medicago* plant organs: nodule (SRX099057), seed pod (including seeds, SRX099058), 4-week blade (SRX099059), flower (SRX099061), 4-week root (SRX099062), all root system (SRX2943065, SRX2943064, SRX2943063) and all shoot system (SRX2943062, SRX2943058). Raw read files were mapped against the *Medicago* transcriptome version 5 (https://Medicago.toulouse.inra.fr/MtrunA17r5.0-ANR/) and quantified as counts using Salmon algorithm^25^. Counts were normalized to corresponding library sizes (equivalent to count per million, CPM) then length of transcripts (Transcript per million, TPM) and displayed as TPM in our study.

## Supplementary Information

Supplementary Figure S1.

Supplementary Figure S2.

Supplementary Figure S3.

Supplementary Figure S4.

Supplementary Figure S5.

Supplementary Figure S6.

Supplementary Figure S7.

Supplementary Table S1.

Supplementary Table S2.

Supplementary Table S3.

Supplementary Table S4.

Supplementary Table S5.

Supplementary Table S6.

Supplementary Table S7.

## Data Availability

All data generated or analyzed during this study are included in this published article (and its supplementary information files).
