# Supplementary material for: Genome-wide association study identified candidate genes for seed size and seed composition improvement in M. truncatula
Source: Sci Rep. 2021 Feb 19;11:4224. doi: 10.1038/s41598-021-83581-7 (PMC7895968; doi:10.1038/s41598-021-83581-7)
Supplement: Supplementary file 1 — Supplementary Figure S1. [file 41598_2021_83581_MOESM1_ESM.pdf]

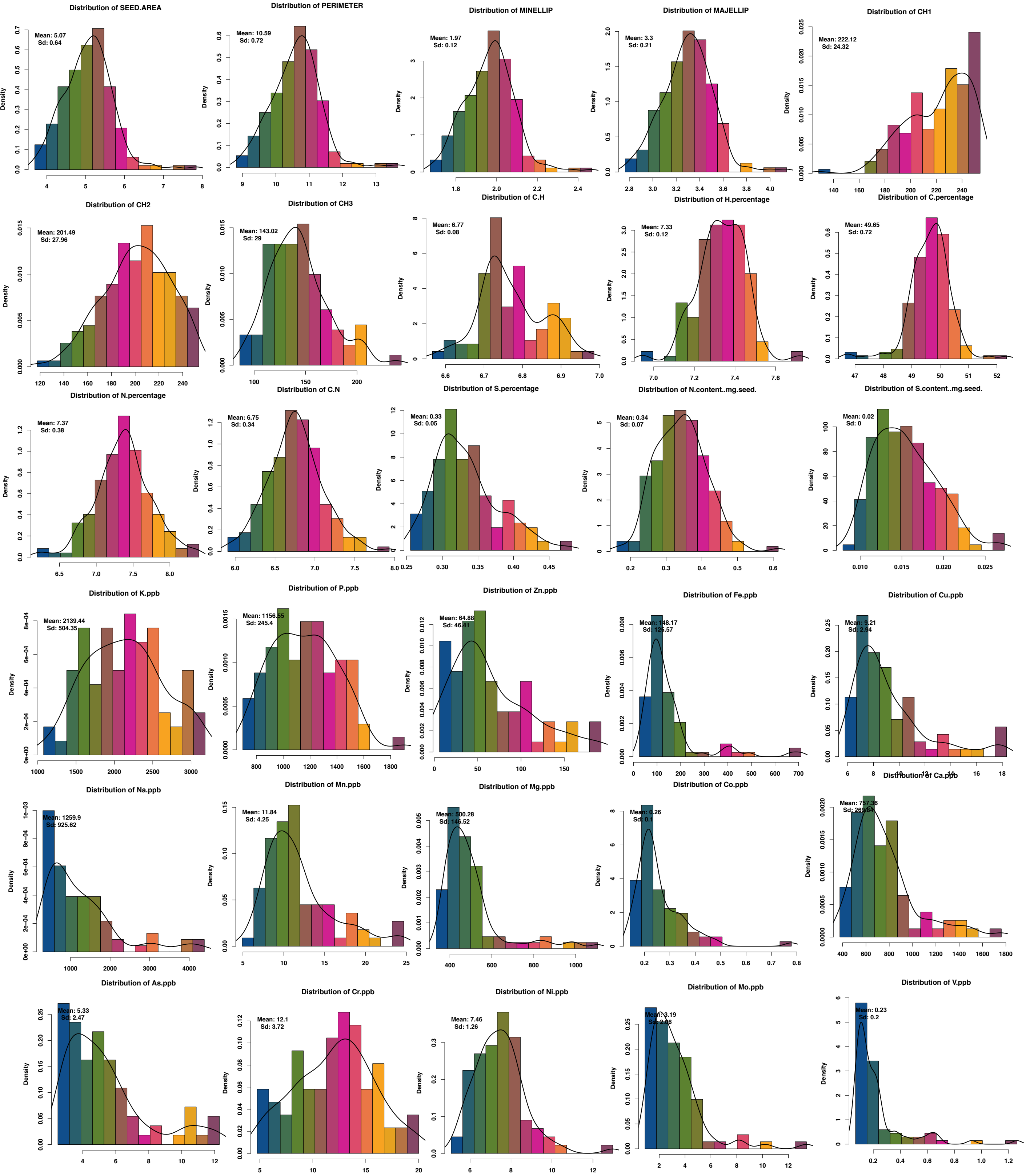

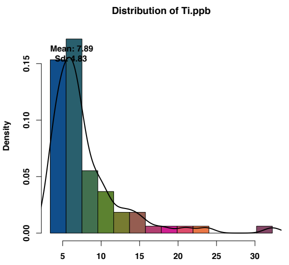

<u>Supplementary figure S1</u>: Distribution histograms of analyzed seed phenotypes in different Medicago accessions. Corresponding distribution curves are indicated on histograms
