# Supplementary material for: Genome-wide association study identified candidate genes for seed size and seed composition improvement in M. truncatula
Source: Sci Rep. 2021 Feb 19;11:4224. doi: 10.1038/s41598-021-83581-7 (PMC7895968; doi:10.1038/s41598-021-83581-7)
Supplement: Supplementary file 2 — Supplementary Figure S2. [file 41598_2021_83581_MOESM2_ESM.pdf]

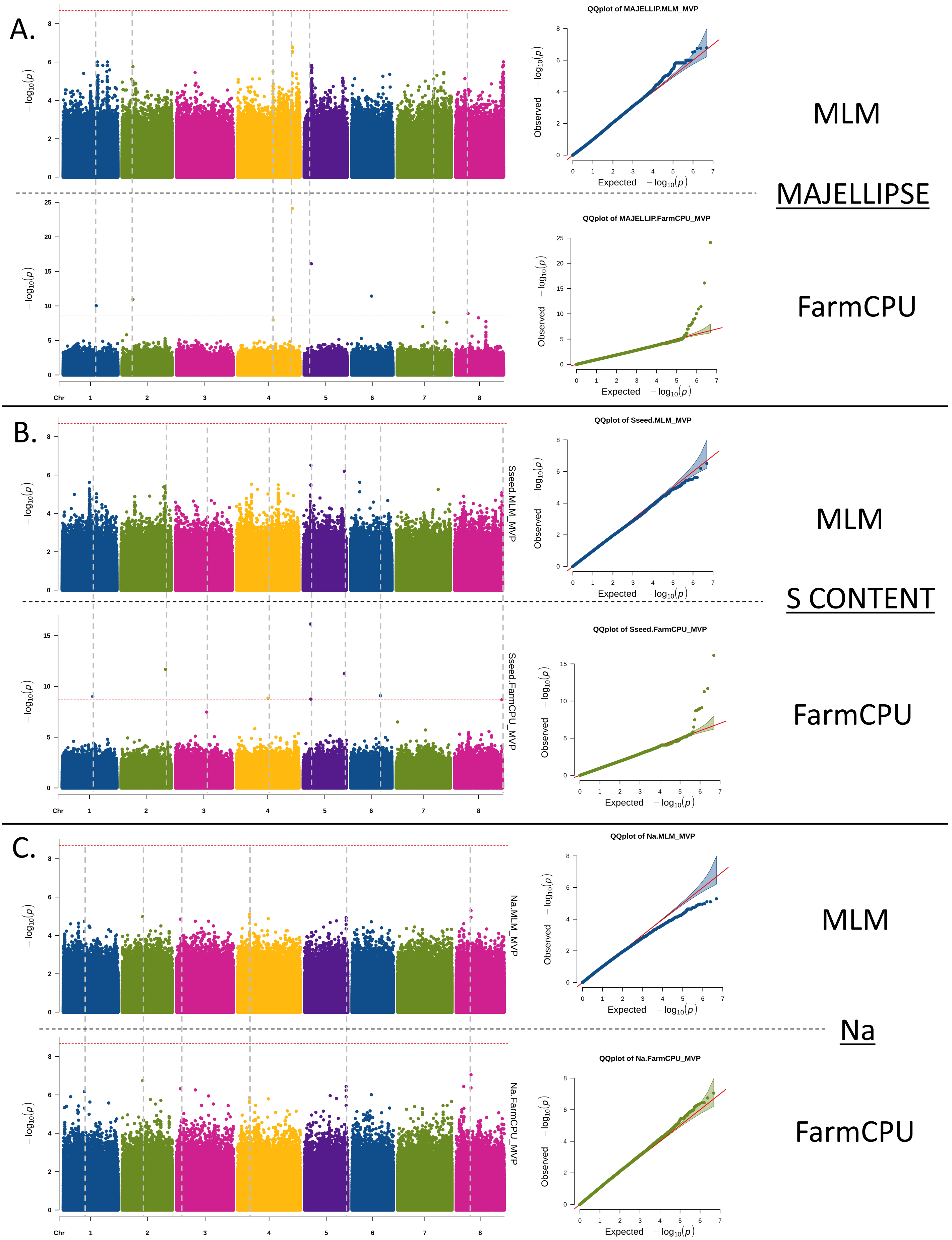

Supplementary figure S2: Manhattan plots obtained using two genome-wide association algorithms: a single locus mixed linear model (EMMA) and a multi-locus mixed model (FarmCPU) with corresponding QQ plots from three phenotypes: A. seed Length, B. sulfur content per seed and C. Na concentration.
