# Supplementary material for: Genome-wide association study identified candidate genes for seed size and seed composition improvement in M. truncatula
Source: Sci Rep. 2021 Feb 19;11:4224. doi: 10.1038/s41598-021-83581-7 (PMC7895968; doi:10.1038/s41598-021-83581-7)
Supplement: Supplementary file 7 — Supplementary Figure S7. [file 41598_2021_83581_MOESM7_ESM.pdf]

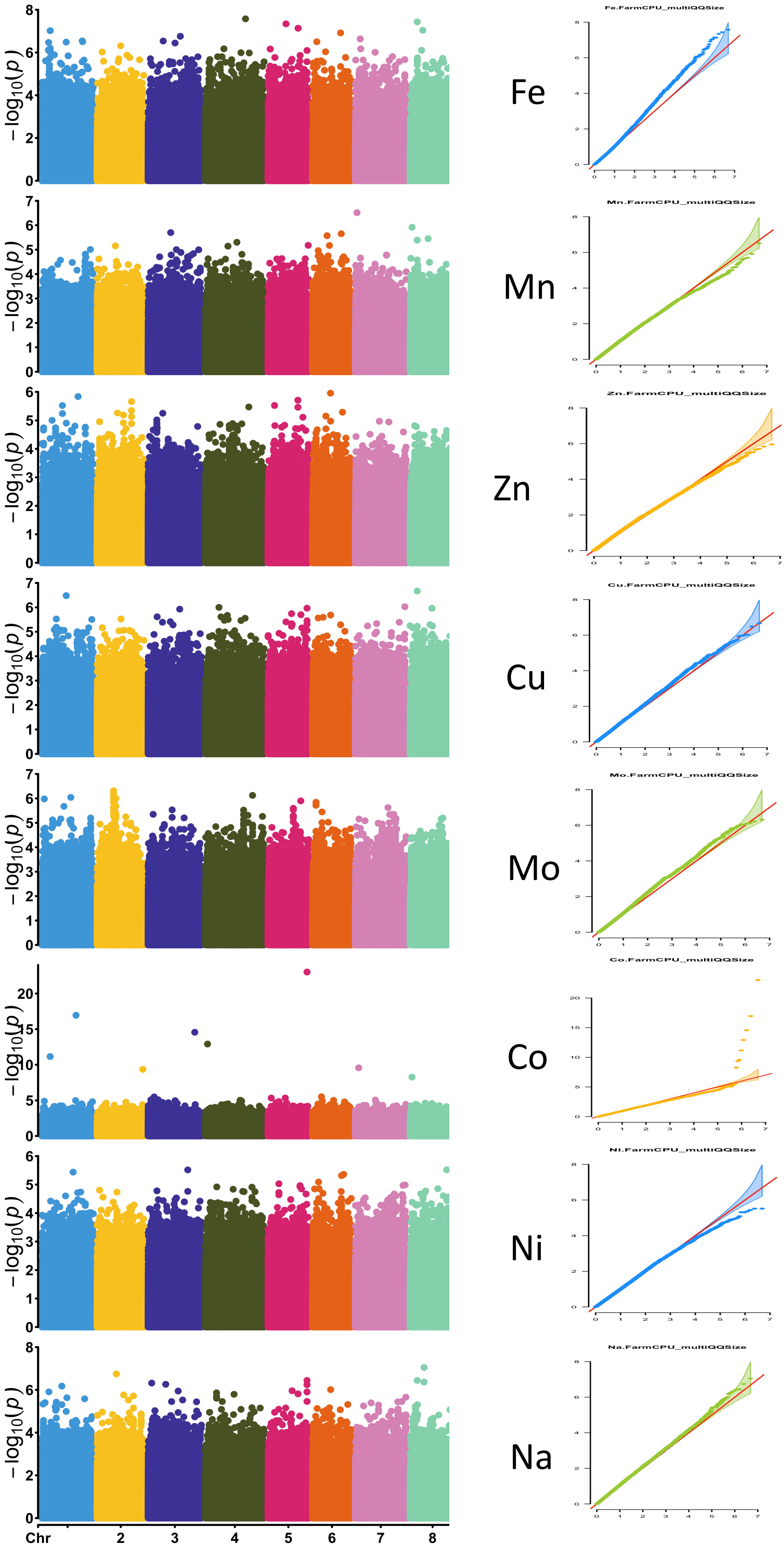

Supplementary figure S7: Manhattan plots obtained using multi-locus model (FarmCPU) with corresponding QQ plots from seed micro-element phenotypes.
